# A Systematic Review of Stress-Related Work and Missed Nursing Care Among Clinical Nurses

**DOI:** 10.3390/healthcare14030304

**Published:** 2026-01-26

**Authors:** Yetty Mardelima Uli Pakpahan, Maria Komariah, Hana Rizmadewi Agustina

**Affiliations:** 1 Master of Nursing Program, Faculty of Nursing, Universitas Padjadjaran, Sumedang 45363, Indonesia; yetty24001@mail.unpad.ac.id; 2 Department of Fundamental Nursing, Faculty of Nursing, Universitas Padjadjaran, Sumedang 45363, Indonesia; hana.rizmadewi@unpad.ac.id

**Keywords:** work stress, missed nursing care, burnout, nurse, patient safety, clinical nurses

## Abstract

**Background:** Missed nursing care (MNCs) is a global issue with the potential to threaten patient safety and is often associated with a stressful work environment. Although stress-related work among clinical nurses is associated with MNCs, the correlation remains limited. **Objective:** This systematic review aimed to assess and synthesize available scientific evidence regarding the correlation between stress-related work among clinical nurses and the incidence of MNCs in hospital settings. **Methods:** This review was conducted according to the PRISMA guidelines. The PubMed, Scopus, and EBSCO databases were systematically searched for articles published between January 2014 and June 2025. Primary studies with quantitative, qualitative, or mixed-methods designs that examined the relationship between stress-related work and MNCs among hospital nurses were included. The data obtained were extracted and analyzed using thematic approaches. **Results:** A total of 244 articles were identified from the three databases. Seven studies, conducted in different countries met the inclusion criteria. All studies used cross-sectional designs. The results showed that most study reported stress-related work, emotional fatigue, and burnout were significantly positively related to the frequency of MNCs (*p* < 0.05). The most frequently missed types of nursing care include monitoring vital signs, skin/wound care, and oral care. **Conclusions:** The evidence suggests that stress-related work among nurses has significant potential to predict MNCs. Interventions that focus on mitigating work stress by improving the work environment and optimizing workload are crucial for improving quality of care and patient safety.

## 1. Introduction

Missed nursing care (MNCs) is a significant challenge that directly affects patient safety and overall health care quality. The reported rates of MNCs vary considerably across healthcare settings, ranging from approximately 7% to 98% in hospitals and 16% to 86% in acute care hospitals. Key factors consistently contribute to MNCs in different settings [1,2]. These include unexpected surges in patient volume, insufficient numbers of nursing staff, and situations requiring immediate patient intervention [1,2]. The consequences for MNCs extend across several dimensions. Patients experience prolonged hospital stay and an increased risk of adverse events [3,4,5]. MNCs may affect public trust in hospitals. Additionally, the economic burden associated with MNCs may be substantial [6]. These conditions also increase healthcare systems expenditures annually through increased care requirements [7].

Occupational stress among nurses has emerged as a critical concern in the healthcare systems worldwide. Multiple contributing factors have been identified, including excessive workload, emotional strain, and increasingly complex ethical dilemmas. These challenges have intensified significantly since the onset of the COVID-19 pandemic [8,9]. The correlation between occupational stress and MNCs can be explained using the Job Demands-Resources model [10]. According to this framework, high job demands that are not balanced with adequate resources lead to energy depletion and exhaustion, suggesting that high job demands, when not matched with adequate resources, lead to exhaustion and diminished performance [10,11]. This imbalance ultimately contributes to MNCs [10,11]. The conservation of resources theory offers another perspective [12]. According to this framework, nurses experiencing stress tend to prioritize direct care activities, such as medication administration [12]. Cognitive load theory provides additional insight into this phenomenon. Excessive stress increases nurses’ cognitive burden, thereby impeding their ability to effectively manage multiple responsibilities [13]. This overload can result in MNCs [13].

Additionally, work-related stress, including burnout and job dissatisfaction, has been shown to be significantly associated with increased instances of MNCs. Burnout, as a form of chronic stress, impairs nurses’ ability to effectively complete tasks, contributing to MNCs that are essential [14]. Evidence has demonstrated a significant correlation between occupational stress and MNCs, with burnout, particularly emotional exhaustion, identified as a major contributor to missed care in nursing settings [14]. Burnout, as a form of chronic stress, not only leads to emotional exhaustion but also exacerbates job dissatisfaction, which in turn increases the likelihood of MNCs [15]. These findings highlight the crucial role of stress and burnout in mitigating missed care and improving patient safety.

From a nursing management perspective, understanding the relationship between occupational stress and MNCs represents a strategic priority for achieving quality and safety standards, rather than merely a human resource concern [16]. Although nurses’ work stress is widely believed to contribute to MNCs, previous studies have not shown consistent results. Evidence shows no significant direct correlation, while others identify stress as an important mediator between workload and missed care [17]. Few limited systematic reviews have comprehensively integrated psychological factors (such as work stress, burnout, and emotional intelligence) with MNCs across nursing service units [18,19]. This systematic review aimed to assess the correlation between occupational stress and MNCs. Additionally, it seeks to provide evidence-based recommendations for organizational strategies that improve both nurses’ well-being and patient safety [20].

## 2. Methods

### 2.1. Study Design

This review employs a systematic review approach that follows the Preferred Reporting Items for Systematic Reviews and Meta-Analyses (PRISMA) guidelines [21]. Adherence ensured optimal transparency and systematic reporting throughout the review. The review question of the study asks whether stress-related work is associated with MNCs among clinical nurses?

### 2.2. Eligibility Criteria

The inclusion criteria for this review were established using the Population, Exposure, and Outcome (PEO) framework to ensure relevance and appropriateness in article selection. The population in this review consisted of clinical nurses, defined as registered or staff nurses working in hospitals. Exposure to this review is stress-related work, defined as occupational stress including job stress, burnout, compassion fatigue, and emotional exhaustion. The outcomes of this review were MNCs, defined as unfinished care, care omissions, nursing care rationing, or implicit rationing. The outcome was measured using a report in the form of an event. Additionally, the context of this review included all hospital settings, including inpatient units, intensive care units, and other service units worldwide.

The exclusion criteria were as follows: non-primary research (e.g., review, letter, editorial), articles published in non-English languages, and articles published before January 2014. Additionally, we excluded studies that did not include full-text data.

### 2.3. Search Strategy

The literature search strategy for this review was comprehensively designed to ensure optimal identification of all relevant studies. The search was conducted across three major electronic databases, PubMed, Scopus, and EBSCO, and covered studies published between 2014 and 2025. The search was conducted on 7 October 2025. The primary keywords encompassed terms related to nurses, occupational stress, and MNCs. These terms were combined using Boolean operators and adapted to the specific characteristics of each database. Keywords were further expanded using synonyms and relevant Medical Subject Headings (MeSH) to enhance the comprehensiveness of the search. The complete search string and keyword used for each database are listed in Table A1. No gray literature was included in this review.

### 2.4. Study Selection

Following duplicate removal, all identified articles underwent a two-stage screening process. In the first stage, two reviewers (YM and MK) independently examined the article titles and abstracts based on predetermined eligibility criteria. Articles deemed relevant were subjected to a full-text review. In the second stage, both reviewers independently assessed article eligibility based on complete text content. If disagreements arise between the two reviewers (YM and MK) at either stage, these issues will be resolved through discussion until consensus is reached. If consensus remained unattainable, a third reviewer (HRA) was involved in the final decision. This approach was implemented to ensure that the study selection process proceeded in an objective and transparent manner.

### 2.5. Study Extraction, Risk of Bias, and Analysis

Data extraction was performed using a standardized form. Three reviewers (YM, MK, and HRA) independently reviewed the included studies throughout the form. The data included key publication characteristics (authors, publication year, country of origin, study design, sample characteristics (e.g., size, gender distribution, mean age, and years of care experience), hospital and care unit setting, measurement tools of exposure and outcome, and correlation findings (r value and *p*-value). All reviewers (YM, MK, HRA) independently assessed the quality of the included studies using the Joanna Briggs Institute (JBI) critical appraisal tools for cross-sectional studies (JBI, Adelaide, Australia) [22]. Descriptive analysis was also conducted. The findings were presented in a descriptive format and supplemented with summary tables.

## 3. Results

### 3.1. Study Selection Results

**Figure 1:**
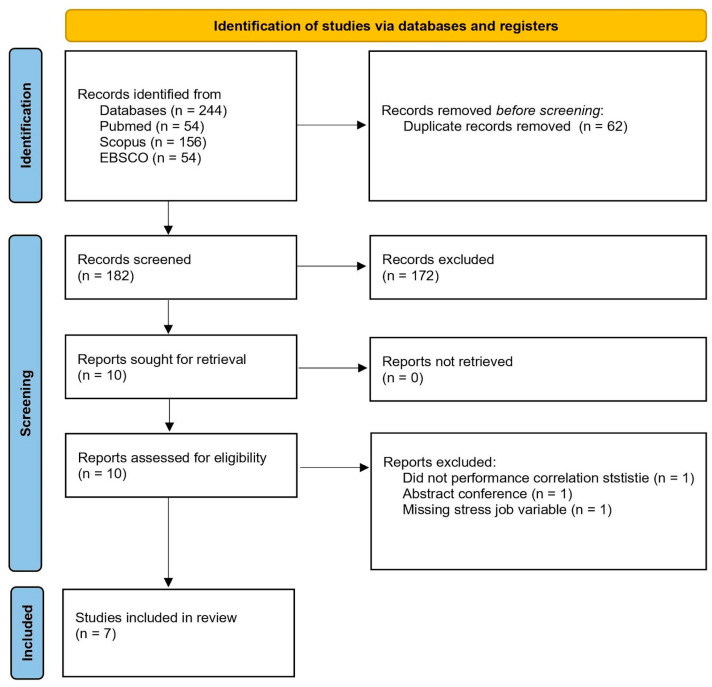
Flow diagram of study selection process, the diagram adapted by [21].

The initial database search identified a total of 244 records from PubMed (n = 54), Scopus (n = 156), and EBSCO (n = 54). Prior to screening, 62 duplicate records were removed, leaving 182 records for title and abstract screenings. During the screening stage, 172 records were excluded because they were irrelevant to the research topic. Subsequently, 10 reports were retrieved, all of which were successfully obtained and assessed for eligibility. Of these, three reports were excluded for the following reasons: one did not perform correlation analysis, one was a conference abstract, and one lacked the job stress variable. As a result, seven studies met the inclusion criteria and were included in the final review [14,23,24,25,26,27,28]. The study selection process is illustrated in Figure 1.

### 3.2. Characteristics of Included Studies

Seven studies were included in the present review. The studies were conducted in Turkey, South Korea, China, the United States, and Saudi Arabia. All the included studies employed a cross-sectional design, with several explicitly described as descriptive cross-sectional studies. The sample size ranged from 140 participants to 706 participants. In all the studies, the nursing workforce was predominantly female. The number of male participants ranged from 3 to 176, whereas female participants ranged from 122 to 637 per study. The mean age of the participants, where reported, ranged from 29.32 to 49.1 years. Information regarding years of professional experience was inconsistently reported across studies; reported mean values ranged from 6.72 to 16.6 years, while several studies categorized experience levels or did not specify this variable.

The studies were conducted in various healthcare settings, including private and public hospitals, secondary and tertiary hospitals, specialized hospitals, government hospitals, and nursing homes. One study was conducted exclusively in nursing homes, whereas the remaining studies were hospital-based. The care unit settings varied across studies and included outpatient chemotherapy units, inpatient cancer units, intensive care units, emergency departments, pediatric units, and mixed or unspecified clinical settings. The detailed characteristics of the included studies are presented in Table 1.

### 3.3. Risk of Bias

The overall methodological quality was generally good across the six included studies, with JBI scores ranging from 6/8 to 8/8 (Table 2). All studies clearly defined the inclusion criteria and provided detailed descriptions of the study participants and settings (J1–J2). Valid and reliable instruments were consistently used to measure exposure and outcomes (J3, J4, and J7), and appropriate statistical analyses were applied across studies (J8), supporting the internal consistency of the findings. However, some methodological limitations related to confounding factors were identified. While several studies identified potential confounders (J5) and reported strategies to address them (J6), others did not adequately identify confounding variables or failed to clearly describe the methods for controlling them. In particular, the limited use of multivariate analyses and incomplete adjustment for demographic and contextual factors may have resulted in residual confounding. These methodological variations should be considered when interpreting the associations reported across studies as they may influence the strength and generalizability of the findings.

### 3.4. Rate of Stress-Related Work and MNCs Among Nurses

Across the included studies, a variety of instruments were used to measure job or work-related stress, and the reported mean stress levels varied widely. Sarubudak et al. [23] assessed stress using burnout subscales measuring helplessness and burnout in combination with the MNCs survey, although no mean stress value was reported. Park and Kim [24] measured job stress using a modified 32-item Kim and Gu scale and reported a mean stress score of 3.54 (SD 0.61). Wang et al. [25] employed an occupational stress scale developed by the authors, and reported a mean stress score of 3.89 (SD 0.34). White assessed emotional exhaustion using the emotional exhaustion subscale of the Maslach Burnout Inventory but did not report a mean stress score. Topal et al. [26] measured stress using the Moral Distress Scale–Revised for pediatric nurses and reported a relatively high mean score of 99.35 (SD 57.74). Alshammari et al. [27] used the Occupational Fatigue Exhaustion/Recovery Scale (OFER-15) alongside the MISSCARE instrument, and reported a mean stress score of 43.8 (SD 13.5). Jia et al. [28] assessed fatigue-related stress using the Fatigue Short Scale (C-CF-Short Scale) together with the MNCs Questionnaire, reporting a mean stress score of 51.17 (SD 26.64).

### 3.5. The Correlation Between Work Stress and Missed Nursing Care (MNCs)

The association between work-related stress and MNCs varied across the included studies (Table 3). Most studies have demonstrated statistically significant relationships [14,24,25,28]. Wang et al. [25] reported a significantly strong positive correlation between occupational stress and MNC (*r* = 0.52, *p* < 0.001). Similarly, Jia et al. [28] and Park and Kim [24] identified a significant moderate association between perceived stress and MNCs (*r* = 0.31, *p* < 0.05; and *r* = 0.213, *p* > 0.01, respectively). Moreover, White et al. [14] found that higher levels of emotional exhaustion were significantly associated with an increased likelihood of MNCs, as indicated by an odds ratio (OR) of 5.53 (95% CI: 2.79–10.96, *p* < 0.001). However, three studies reported no significant association between burnout and MNCs (*p* > 0.05).

## 4. Discussion

This systematic review showed that most studies reported a positive correlation between work-related stress and MNCs, with correlation coefficients 0.213–0.52. Regression analyses confirmed stress as a significant predictor of MNCs frequency, primarily driven by organizational factors including excessive workload, inadequate staffing, and time pressure. The most frequently omitted care activities included vital sign monitoring, skin/wound care, and oral care. This study found that the association between work-related stress and MNCs varied across the included studies, with several reporting statistically significant relationships. Higher levels of occupational and perceived stress, particularly emotional exhaustion and workload-related stressors, are associated with increased MNCs. These findings are consistent with those of previous systematic reviews by Monalisa et al. [29] and Rahmah et al. [19] who identified burnout and excessive workload as key contributors to MNCs.

While earlier reviews primarily emphasized structural factors, such as staffing and organizational constraints, the study highlights the role of psychological stressors in MNCs. Drawing on the Job Demands–Resources Model and Cognitive Load Theory, the findings suggest that emotional exhaustion and cognitive overload may impair nurses’ ability to prioritize and deliver comprehensive care, especially in non-technical aspects such as communication and patient education [10]. Furthermore, intensified work stress in the post-pandemic context may increase the risk, underscoring the need to address both structural and psychosocial stressors.

The association between work-related stress and MNCs explains the theoretical framework. The job demands–resources model clarifies how chronic demands deplete nurses’ cognitive and emotional resources, leading to exhaustion and performance impairment [10,30], directly aligning with our finding that workload is a primary stressor. Cognitive Load Theory further explains how stress overwhelms working memory, compromising nurses’ capacity for complex clinical reasoning and multi-tasking [31,32], and the conservation of resources theory elucidates the subsequent rationing of psychosocial care elements, such as communication and patient education [12,31], precisely the care components most vulnerable to MNCs in our analysis. These mechanisms collectively create a pathway through which organizational stressors systematically translate into MNCs.

### 4.1. Implications

Although several studies have reported statistically significant correlations between work-related stress and MNCs, the magnitude of these associations was generally modest (e.g., correlation coefficients around 0.3–0.4). In the context of complex, multifactorial clinical environments, such effect sizes should be interpreted cautiously [19]. A correlation of this magnitude does not indicate that work-related stress is the sole or dominant determinant of MNCs; rather, it suggests that stress is a contributing factor, alongside structural and organizational influences such as staffing levels, patient acuity, workflow design, and leadership support [19,29]. Nevertheless, even modest associations may have meaningful clinical implications when they operate across a large nursing workforce and over sustained periods. For example, persistent emotional exhaustion or elevated workload-related stress may incrementally impair nurses’ capacity to consistently deliver non-technical aspects of care, such as patient education and communication, which are particularly vulnerable to MNCs and may affect patients’ adverse events [18]. Thus, although the observed correlations are moderate rather than strong, they remain clinically relevant and support the need for interventions that address both psychological stressors and broader system-level factors.

Several organizational-level interventions have been proposed to mitigate nurses’ work-related stress, including workload adjustments and participatory approaches involving nurses in organizational processes. Organizational environments with positive workplace cultures have been shown to reduce both stress levels and MNCs incidents [33,34]. Additionally, emotional labor strategies, particularly deep acting, can help mitigate the impact of compassion fatigue on MNCs [28]. Other contributing factors also influence the prevalence of MNCs. These include inadequate staffing levels, high patient volumes, and nurses’ experience. Nurses with less work experience tend to report higher MNC rates [35].

Implementing system-level interventions based on the Job Demands–resources framework is crucial for disrupting the stress MNCs cycle. Transitioning to acuity-based nurse-to-patient ratios, such as 1:4 in medical-surgical units, addresses the frequent shortfall in required RN hours, which contributes to approximately 21% of missed care episodes [35,36]. Complementary resources, such as dedicated support staff and integrated health technologies, reduce cognitive overload and improve care quality [37,38]. Additionally, fostering transformational leadership and structured peer support programs create supportive environments that alleviate occupational stress [13]. Policymakers must enforce evidence-based staffing standards and regulatory mandates to enhance patient safety, sustain nursing workforce, and mitigate adverse events and operational costs [13,14,39].

### 4.2. Strength and Limitations of the Study

Although this study comprehensively explains the correlation between stress-related work and MNCs, several limitations must be considered when interpreting the findings. The predominance of cross-sectional studies in the included literature prevented definitive conclusions regarding causality in the relationship between work stress and missed nursing care. Significant heterogeneity was observed in the methodological approaches, measurement instruments, and operational definitions of both key variables across studies, complicating the direct comparison of the findings. The exclusive focus of quantitative studies may overlook important subjective experiences and contextual factors that influence nurses’ decisions regarding care prioritization. The relatively small number of included studies, resulting from our restriction to English-language publications and specific timeframes, along with the underrepresentation of healthcare settings in regions such as Latin America, Africa, and the Middle East, may limit the generalizability of our findings across different global healthcare contexts and cultural environments. Moreover, this review included a relatively small number of studies, which limits the robustness of the conclusions. With a restricted evidence base, the observed associations between work-related stress and MNCs may be influenced by individual studies, thus reducing confidence in the consistency and strength of the relationship.

All included studies used a cross-sectional design, which imposes an important constraint on interpretation: the evidence can describe associations, but it cannot establish temporality or support causal inferences. The overall strength of the evidence for a causal relationship is limited, even when statistically significant associations are reported. Although the JBI checklist ratings suggested generally acceptable methodological quality (scores ranging from 6/8 to 8/8), a more detailed inspection indicated that the most recurrent limitation across the body of evidence relates to confounding. Several studies did not clearly identify potential confounders (J5) or report strategies to manage confounding (J6). This is because key contextual factors, such as staffing levels, workload intensity, shift length, unit type, patient acuity, and leadership support, are plausibly associated with both nurses’ stress and the likelihood of missed care. A high workload may simultaneously increase stress and missed care, making the association appear stronger than it would be under adequate adjustment. Conversely, heterogeneity in the unit context may weaken associations in some settings despite a true underlying link. In addition, variability in the stress constructs assessed (e.g., perceived stress, emotional exhaustion, burnout, moral distress, and fatigue) and differences in how MNCs were operationalized likely contributed to inconsistency across findings, with some studies reporting significant associations and others reporting non-significant relationships.

Future research should prioritize longitudinal cohort and intervention studies to clarify the temporal and causal relationships between work-related stress and MNCs. Such designs would allow for a more robust examination of how changes in stress levels influence care delivery over time and enable the evaluation of the effectiveness of stress-reduction or organizational interventions. In addition, there is a need to address geographic, cultural, and health system-level gaps in the current evidence base, as most existing studies originate in Western countries. Expanding research on diverse cultural and healthcare contexts, particularly in low- and middle-income countries, would enhance the generalizability of the findings and provide insights into how contextual factors shape the stress–missed care relationship.

## 5. Conclusions

This systematic review provides evidence that stress-related work among nurses, which manifests as burnout and emotional exhaustion, is a significant predictor of MNCs in hospitals. This systematic review strengthens the evidence that psychological factors are associated with MNCs. By integrating findings across diverse settings, this review clarifies that work-related stress is consistently associated with missed nursing care, particularly in the psychosocial and non-technical aspects of care. However, several issues remain unresolved, including the limited ability to infer causality due to the predominance of cross-sectional designs, substantial heterogeneity in measurement instruments, and underrepresentation of non-Western healthcare contexts. Future longitudinal and interventional studies are needed to strengthen causal inference, improve comparability, and inform evidence-based strategies to reduce missed nursing care.

## Figures and Tables

**Table 1:** Characteristics of included studies.

Author	Country	Study Design	Sample Size	Gender (M/F)	Mean Age (Years)	Years of Care Experience (Years)	Hospital Setting	Care Unit Setting
Sarıbudak et al. [23]	Turkey	Cross-sectional	147	12/135	29.32	6.72	Private and public	Outpatient chemotherapy and inpatient cancer units
Park & Kim [24]	South Korea	Descriptive Cross-sectional	164	3/159	29.8	NA	Secondary and tertiary	All setting
Wang et al. [25]	China	Cross-sectional	706	176/530	30.55	1–5 = 341 nurses 5–10 = 281 nurses >10 = 84 nurses	Tertiary and secondary	NA
White et al. [14]	USA	Cross-sectional	687	50/637	49.1	16.6	Nursing home	Nursing home
Topal et al. [26]	Turkey	Descriptive Cross-sectional	140	18/122	31.50 (SD 6.38)	NA	Secondary	Specialized hospital (Pediatric)
Alshammari et al. [27]	Saudi Arabia	Cross-sectional	183	23/160	NA	<6 = 91 nurses >6 = 92 nurses	Tertiary	Emergency room, pediatric unit, and intensive care unit
Jia et al. [28]	China	Cross-sectional	226	73/153	NA	Not Specified	Not specified	Intensive Care Unit

**Table 2:** Quality assessment of included studies.

Study	J1	J2	J3	J4	J5	J6	J7	J8	Overall
Sarıbudak et al. [23]	Yes	Yes	Yes	Yes	No	Yes	Yes	Yes	7/8
Park & Kim [24]	Yes	Yes	Yes	Yes	No	Yes	Yes	Yes	7/8
Wang et al. [25]	Yes	Yes	Yes	Yes	No	No	Yes	Yes	6/8
White et al. [14]	Yes	Yes	Yes	Yes	Yes	Yes	No	Yes	7/8
Topal et al. [26]	Yes	Yes	Yes	Yes	No	No	Yes	Yes	6/8
Alshammari et al. [27]	Yes	Yes	Yes	Yes	Yes	No	Yes	Yes	7/8
Jia et al. [28]	Yes	Yes	Yes	Yes	Yes	Yes	Yes	Yes	8/8

Note: J1: Were the criteria for inclusion in the sample clearly defined? J2: Were the study participants and settings described in detail? J3: Was exposure measured in a valid and reliable way? J4: Were objective and standard criteria used for the measurement of the condition? J5: Were the confounding factors identified? J6: Were strategies used to deal with the confounding factors stated? J7: Were the outcomes measured in a valid and reliable manner? J8: Was appropriate statistical analysis used?

**Table 3:** Correlation between work stress and missed nursing care.

Author	Measurement Tools	Mean Stress	Type of Missed Care	Correlation Findings	Statistical Values
Sarıbudak et al. [23]	The burnout subscale measures levels of hopelessness and burnout, and the Missed Nursing Care Survey	NA	Vital signs assessed as ordered, monitor intake/output, skin care, mouth care	There is no correlation	*p* < 0.493
Park & Kim [24]	Modified 32 items- Kim and Gu Scale	3.54 (SD 0.61)	Turning patient, feeding, preparing meals, medication administrated, vital sign, monitor intake/output, skin care, mouth care, glucose monitoring, IV line, wound care	There is significant correlation	*r* = 0.213, *p* < 0.01
Wang et al. [25]	Occupational Stress Scale developed by Wang et al.	3.89 (SD = 0.34)	NA	There is significant correlation	*r* = 0.52, *p* < 0.001 *
White et al. [14]	Emotional Exhaustion subscale of the Maslac-Burnout Inventory	NA	Oral hygiene/mouth care, skin care, pain management	There is significant correlation	OR = 5.53 (2.79–10.96) *p* > 0.001
Topal et al. [26]	Moral Distress Scale-Revised Pediatrics Nurses	99.35 SD (57.74)	Mouth care, body hygiene and skin care, pain assessment, monitoring intake/output of solid and liquid	There is no significant correlation	*r* = 0.105 *p* > 0.05 *
Alshammari et al. [27]	MISSCARE and Occupational Fatigue Exhaustion/Recovery Scale (OFER-15)	43.8 (SD = 13.5)	Oral hygiene, assistance with meal, high patient load, patient bathing and skin care	There is no correlation	(r = −0.056, 95% CI: −0.20 to 0.09, *p* = 0.452)
Jia et al. [28]	Missed Nursing Care Questionnaire and Fatigue-Short Scale (C-CF-Short Scale)	51.17 (SD = 26.64)	NA	There is significant correlation	r = 0.351 *p* > 0.05 *

Note: * statistically significant (*p* < 0.05).

## Data Availability

No new data were created or analyzed in this study.

## Abbreviations

The following abbreviations are used in this manuscript:

MNCs	Missed Nursing Cares
PRISMA-ScR	Preferred Reporting Items for Systematic reviews and Meta-Analyses (Systematic Review)
PEO	Population, Exposure, and Outcome
MeSH	Medical Subject Headings
JBI	Joanna Briggs Institute
ICUs	Intensive Care Units
MBI	Maslach Burnout Inventory
BMS	Burnout Measure-Short Version
PSS-10	Perceived Stress Scale
NASA-TLX	NASA Task Load Index
BERNCA-R	Believed to be a survey for missed care/rationing, not explicitly defined in full
PIRNCA	Believed to be a scale for implicit rationing of nursing care, not explicitly defined in full
SEM	Structural Equation Modeling
CFI	Comparative Fit Index
RMSEA	Root Mean Square Error of Approximation
OR	Odds Ratio
CI	Confidence Interval
OFER-15	Occupational Fatigue Exhaustion/Recovery Scale
NA	Not Available

## Appendix A

**Table A1:** Search strategy.

Database	Keyword	Retrieved
Pubmed	(“nurses”[MeSH Terms] OR “nurses”[Title/Abstract] OR “nursing staff”[Title/Abstract] OR “registered nurses”[Title/Abstract]) AND (“occupational stress”[MeSH Terms] OR “work stress”[Title/Abstract] OR “job stress”[Title/Abstract] OR “workplace stress”[Title/Abstract] OR “burnout, professional”[MeSH Terms] OR “workload”[Title/Abstract]) AND (“Missed nursing care”[Title/Abstract] OR “missed care”[Title/Abstract] OR “unfinished care”[Title/Abstract] OR “care left undone”[Title/Abstract] OR “nursing care omission”[Title/Abstract] OR “implicit rationing”[Title/Abstract]) AND (“hospital”[MeSH Terms] OR “hospital”[Title/Abstract] OR “acute care”[Title/Abstract] OR “inpatient”[Title/Abstract])	54
Scopus	TITLE-ABS-KEY (nurse* OR “nursing staff” OR “registered nurse*” OR “hospital nurse*”) AND (“occupational stress” OR “work stress” OR “job stress” OR “workplace stress” OR “burnout” OR “workload”) AND (“Missed nursing care” OR “missed care” OR “unfinished care” OR “care left undone” OR “nursing care omission” OR “implicit rationing”) AND (hospital* OR “acute care” OR inpatient OR “clinical setting” OR ward)	156
EBSC0	(“nurse*” OR “registered nurse*” OR “staff nurse*” OR “clinical nurse*”) AND (“job stress” OR “work stress” OR “occupational stress” OR “workload stress” OR “stress at work” OR “burnout” OR “emotional exhaustion”) AND (“Missed nursing care” OR “care left undone” OR “unfinished nursing care” OR “rationing of nursing care” OR “implicit rationing” OR “omitted nursing care”) AND (“hospital” OR “acute care” OR “inpatient ward” OR “ICU” OR “emergency department”)	34
